# Loss of SHP-1 in CD11c^+^ cells impairs anti-tumor immunity

**DOI:** 10.3389/fimmu.2026.1710547

**Published:** 2026-04-21

**Authors:** Miguel Galán, Elena Hernández-García, Pablo Munné, Ana Redondo-Urzainqui, Francisco J. Cueto, Jon Sicilia, Sergio Callejas Alejano, M. Ascensión Rey-Martín, Ana Dopazo, David Sancho, Salvador Iborra

**Affiliations:** 1 Centro Nacional de Investigaciones Cardiovasculares Carlos III (CNIC), Madrid, Spain; 2 Escuela de Doctorado, Universidad Autónoma de Madrid, Madrid, Spain; 3 Fundación Inmunotek, Alcalá de Henares, Spain; 4 Department of Immunology, Ophthalmology and Ear, Nose and Throat (ENT), School of Medicine, Universidad Complutense de Madrid, Madrid, Spain

**Keywords:** CD8^+^ T cell response, conventional type 1 dendritic cells, macrophages, SHP-1, tumor rejection

## Abstract

**Background:**

Tyrosine kinases and phosphatases regulate protein phosphorylation and maintain cellular homeostasis. The phosphoprotein tyrosine phosphatase SHP-1 (encoded by the *Ptpn6* gene) has been proposed as an immune checkpoint in CD8^+^ T cells in preclinical models, yet its pharmacological inhibition has shown no efficacy against tumor growth in clinical trials. This suggests that SHP-1 may play opposing roles in different cell types within the tumor microenvironment. Here, we investigated the effect of depleting SHP-1 in CD11c^+^ cells on the anti-tumoral response.

**Methods:**

To dissect the specific role of SHP1 in CD11c^+^ antigen-presenting cells, or specifically in conventional type 1 dendritic cells (cDC1s) or macrophages, we subcutaneously inoculated different tumors in *ItgaxΔPtpn6*, *Xcr1*Δ*Ptpn6* and *Lyz2*Δ*Ptpn6* mice, respectively. Tumor growth and survival were monitored, and immune infiltrates were analyzed using flow cytometry or scRNA-seq.

**Results:**

Tumor rejection was impaired when SHP-1 was depleted in CD11c^+^ cells, as well as in XCR1^+^ or Lyz2^+^ cells. scRNA-seq analysis revealed that both tumor-associated macrophages and cDC1s exhibited downregulation of interferon response pathways in tumor-bearing *ItgaxΔPtpn6* mice compared with controls. Reduced MHC-II expression in tumor-associated macrophages was validated by flow cytometry, supporting impaired antigen presentation in these cells, whereas cDC subsets displayed heterogeneous alterations in co-stimulatory marker expression rather than a defect. Consistent with these findings, flow cytometry analysis showed that *ItgaxΔPtpn6* mice injected with MC38 tumor and treated with anti-PD1 displayed a reduction in CD8^+^ IFN-γ cells in comparison with *Ptpn6 ^f/f^* littermates.

**Conclusions:**

These results show that SHP-1 depletion in CD11c^+^ cells impairs anti-tumor immunity and suggest that both cDC1s and macrophages contribute to this effect.

## 1 Introduction

Dendritic cells (DCs) are professional antigen-presenting cells with a pivotal role in initiating and shaping anti-tumor immunity. Among them, conventional type 1 dendritic cells (cDC1s; CD11c^+^ MHCII^+^ XCR1^+^ CD11B^-^) are uniquely effective at cross-presenting cell-associated antigens on MHC class I, thereby priming naïve CD8^+^ T cells and driving cytotoxic CD8^+^ T lymphocyte (CTL) responses against tumors (1–4). After capturing tumor-associated antigens within the tumor microenvironment (TME), cDC1s mature and upregulate costimulatory molecules and the chemokine receptor CCR7, which enables their migration to tumor-draining lymph nodes (tdLNs). There, they activate tumor-specific naïve T cells (5–8). CCR7^+^ DCs, commonly referred to as migratory DCs, exhibit enhanced expression of co-stimulatory molecules (e.g.: CD80, CD86, CD40) and are particularly effective at priming tumor-specific CD8^+^ T cells (5, 9). The functionality of CCR7^+^ DCs is closely linked to a competent interferon gamma (IFN-γ) signaling axis, which promotes the maturation and antigen-presenting capacity, critical processes for the recruitment and activation of effector T cells (10).

By contrast, tumor-associated macrophages (TAMs) represent a dominant myeloid population within solid tumors that frequently promote tumor progression and immune evasion (11, 12). TAMs are highly plastic and adapt their phenotypes in response to tumor-derived cues, hypoxia, and metabolic reprogramming, exhibiting a continuum of heterogeneous states. Distinct TAM subsets, such as lipid-associated (LAMs), angiogenic TAMs, or interferon-responsive macrophages, exert divergent effects on antitumor immunity and therapy response (13). In most solid tumors, TAMs promote angiogenesis and expression of immunosuppressive cytokines (e.g.: IL-10, TGF-β), which inhibit adaptive immunity and limit responsiveness to checkpoint blockade (14). IFN-γ counteracts this suppressive state by promoting pro-inflammatory polarization, enhancing antigen presentation, and supporting macrophage tumoricidal capacity. Conversely, impaired IFN-γ signaling in TAMs has been associated with immune suppression and poor responsiveness to immunotherapy (15, 16).

Given the central role of cDC1s and TAMs in orchestrating IFN-γ–dependent antitumor responses, it is critical to understand the intracellular brakes that limit their function. One such brake could be the Src homology region 2 domain-containing phosphatase-1 (SHP-1), encoded by the *Ptpn6* gene and broadly expressed in hematopoietic cells (17). SHP-1 is a cytoplasmic tyrosine phosphatase that negatively regulates signaling pathways downstream of various immune receptors (18). In DCs and macrophages, SHP-1 affects activation of downstream signaling cascades following stimulation of receptors such as toll like receptors (TLRs), cytokine receptors, and Fc receptors (19–22). In T cells, SHP-1 sets the activation threshold by dephosphorylating proximal signaling molecules, thereby attenuating T cell receptor (TCR) signaling and preventing hyperactivation (23, 24). Because of its inhibitory role, SHP-1 has emerged as a potential target in cancer immunotherapy. Inhibition or knockdown of SHP-1 in T cells enhances antitumor responses by expanding the pool of tumor-reactive T cells (25, 26). Notably, SHP-1 inhibition synergizes with immune checkpoint blockade therapies such as anti-PD-1 and anti-CTLA-4, helping to overcome T cell exhaustion and resistance mechanisms in the TME. Consequently, pharmacological inhibition of SHP-1 is being explored as a strategy to boost anti-tumor immunity, particularly in combination with checkpoint inhibitors. However, clinical development of SHP-1 inhibitors has been limited by disappointing efficacy and concerns about unintended immunosuppression or myeloid dysfunction (27, 28).

Contrary to our expectations, our results show that SHP-1 deletion in CD11c^+^ cells accelerates tumor growth, revealing a detrimental effect on anti-tumor immunity. Single-cell RNA sequencing (scRNA-seq) identified impaired IFN-γ response pathways in both cDC1 and macrophages, while flow cytometry confirmed reduced CD8^+^ IFN-γ^+^ T cells in tumor-bearing mice, consistent with defective priming and effector activity.

These findings provide mechanistic insight into SHP-1 regulation of myeloid cell–mediated anti-tumor immunity. They also help explain the limited efficacy of SHP-1 inhibitors in clinical trials. Rather than augmenting responses, SHP-1 loss in CD11c^+^ cells compromises DC and macrophage support of CD8^+^ T cells, highlighting the need for precise cell-specific targeting strategies.

## 2 Materials and methods

### 2.1 Mouse strains

Mice were bred and housed in groups of 5 animals per cage at the CNIC under specific pathogen-free conditions. To study the function of SHP-1 (codified by *Ptpn6* gene) in different immune cells, we crossed the B6.129P2-Ptpn6tm1Rsky/J mice (The Jackson Laboratory, strain 008336) kindly donated by Clifford Lowell (UCSF) (22) with *Itgax^Cre^* mice (The Jackson Laboratory, strain 008068) kindly donated by Boris Reizis (NYU) (29) or *Xcr1^Cre^* mice kindly donated by Bernard Malissen (CNRS) (The Jackson Laboratory, strain 035435) (30) or *Lyz2^Cre^* mice (The Jackson Laboratory, strain 004781) (31) to generate *ItgaxΔPtpn6, Xcr1*Δ*Ptpn6* and *Lyz2*Δ*Ptpn6* respectively. Only female mice (7–8-week-old at the initiation of experiments) were used and they were maintained on a 12-hour light/dark schedule with ad libitum access to food and water, at 20–24 °C and 45–65% relative humidity. Animal studies were approved by the local ethics committee at CNIC, UAM and Comunidad de Madrid, PROEX 172-19, PROEX 304.8/21 and PROEX 172.5/24. All animal procedures conformed to EU Directive 2010/63EU and Recommendation 2007/526/EC regarding the protection of animals used for experimental and other scientific purposes, enforced by Spanish law under Real Decreto 53/2013.

### 2.2 Tumor cells lines

MC38 (purchased from the ATCC/Kerafast accession ID: CVCL_B288), B16-OVA (a kind gift from L. Chen, Yale University, New Haven, CT) and EG7-OVA cells (EG7, derivative of EL4 were kindly provided by Caetano Reis e Sousa, The Crick Institute, London) tumoral cell lines were cultured at 37 °C and 5% CO_2_ in Dulbecco’s Modified Eagle Medium (Gibco) supplemented with 10% FBS (Sigma-Aldrich), 2 mM L-glutamine (Lonza), penicillin (100 U/ml) (Lonza), streptomycin (100μg/ml) (Loza), non-essential amino acids (Hyclone), 1mM sodium pyruvate (Hyclone), HEPES (10mM) (Hyclone), and β-mercaptoethanol (50μM) (Sigma-Aldrich). All cell lines were tested for the absence of mycoplasma using the MycoAlert PLUS Mycoplasma Detection Kit (Lonza) according to manufacturer’s instructions. Tumor cells were passaged in 5 mM EDTA/phosphate-buffered saline (PBS).

### 2.3 Inoculation of tumor cells and *in vivo* treatments

On the day of injection, tumor cells were washed twice with PBS, and 5×10^5^ cells for MC38 or B16-OVA and 1x10^6^ for EG7 cells in 100 μl PBS were inoculated subcutaneously into the shaved right flank of isoflurane-anesthetized mice. For the MC38 tumor setting, 5, 7, and 9 days after tumor injection, tumor-bearing mice were treated intraperitoneally with 200µg of anti-PD1 (clone RMP1–14 from BioXCell). Tumor size was measured three times per week with an electronic caliper and calculated as the product of the longest diameter and its orthogonal. Mice were euthanized following humane endpoint criteria (pain, necrotic tumor or tumor size bigger than 324 mm^2^).

### 2.4 Tissue digestion and flow cytometry

Tumors and tdLNs were collected at the indicated time points for flow cytometry analysis. Tumors were cut with scissors and digested in 0.5 mg/mL Collagenase IV (Sigma-Aldrich) and 0.2 mg/mL DNase I (Sigma-Aldrich) for 1 hour at 37 °C with agitation. The digested tissues were filtered through a 100μm cell strainer in cold FACS Buffer (PBS, 2.5% heat-inactivated fetal bovine serum (FBS), 2 mM EDTA and 0.01% sodium azide) and red blood cell lysis was performed for 5 minutes at room temperature. Then, the lysis was neutralized with FACS buffer. tdLNs were cut with scissors and digested in HBSS containing 0.25 mg/mL Liberase TL (Roche) and 50 µg/mL DNase I (Sigma-Aldrich) for 15 minutes at 37 °C. The digestion was stopped by adding iFBS, and samples were mechanically dissociated by repeated pipetting. The resulting cell suspensions were filtered through a 40 µm cell strainer and resuspended in antibody staining mix for subsequent flow cytometry staining.

To analyze myeloid populations, cells were washed with PBS and stained for 30 min at 4 °C with LIVE/DEAD™ Fixable Blue Dead Cell Staining Kit, for UV Light Excitation (ThermoFisher). Then, cells were again washed with FACS Buffer and stained for 30 minutes at 4°C in FACS Buffer with anti-CD16/32 (Tonbo Biosciences) and antibody cocktail containing CD45.2-UV805 (Thermo Fisher Scientific # BDB741957), CD11b-UV395 (BD Bioscience #563553), LY6C-eF450 (eBioscence #48-5932-80), XCR1-BV785 (Biolegend #148225), F4/80-FITC (Thermofisher #11-4801-82), CD11c-PE (BD Bioscience #553802), CD86-Pe/Cy7 (eBioscience #25-0862-80), CD80-AF647 (Biolegend #104718) and MHC II-eFluor 780 (eBioscience #47-5321-80). Samples were then washed once with FACS buffer and resuspended in FACS buffer until acquisition. Precision Count Beads (Biolegend) were added to each sample to estimate absolute cell numbers.

To analyze CD8^+^ T cells response, single cell suspensions from tumor and dLN were stimulated with 50 ng/mL phorbol 12-myristate 13-acetate (PMA, Sigma-Aldrich), 1 μg/mL ionomycin (Sigma-Aldrich) for 2 h and then 5 μg/mL brefeldin-A (Sigma-Aldrich) were added to the culture for additional 2 hours at 37 °C and 5% CO_2_. Cells were washed with PBS and stained for 30 min at 4 °C with LIVE/DEAD™ Fixable Blue Dead Cell Staining Kit, for UV Light Excitation (ThermoFisher). Then, cells were again washed with FACS Buffer and stained for 30 minutes at 4°C in FACS Buffer with anti-CD16/32 (Tonbo Biosciences) and antibody cocktail containing CD45.2-UV805 (Thermo Fisher Scientific # BDB741957), CD90.2-V785 (Biolegend #105331), CD8b-PerCP-eFluor710 (eBioscence #46-0083-82), CD4-APC (BD Bioscience #553051), CD44-BV650 (Biolegend # 103049), CD279 (PD-1)-PE (Biolegend #135206). Cells were fixed and permeabilized with Fixation/Permeabilization Solution (Thermo Fisher Scientific) and stained intracellularly for an additional 30 minutes with antibody cocktail containing IFNγ-BV421 (Biolegend # 505830) and Granzyme B-Pe/Cy7 (Biolegend #372214). Samples were then washed once with FACS buffer and resuspended in FACS buffer until acquisition. Precision Count Beads (Biolegend) were added to each sample to estimate absolute cell numbers. All extracellular antibodies were used at 1/200 dilution, while intracellular ones were used at 1/100 dilution. Data acquisition was performed using a FACSymphony (Becton Dickinson) flow cytometer and analyzed with FlowJo software version 10 (TreeStar).

### 2.5 Single-cell RNA sequencing

For single-cell analysis of myeloid tumor infiltrating cells, tumors from five mice per genotype were pooled and processed into single-cell suspensions, as described before, that were subjected to positive selection of CD11c^+^ cells using CD11c MicroBeads UltraPure (Miltenyi Biotec). Briefly, cells were incubated at 4°C for 15 minutes with 100 μL of FACS buffer containing 25 μL of anti-CD11c MicroBeads in the presence of anti-mouse CD16/CD32. Then, cells were washed with 10 mL of FACS buffer, resuspended in 1ml of FACS buffer, and purified with a QuadroMACS™ separator using LS columns (Miltenyi Biotec) following the manufacturer’s instructions. Purified CD11c^+^ cells were stained for 30 minutes at 4°C with an antibody cocktail containing CD11c-FITC (Biolegend #117305). Then, CD11c^+^ cells were sorted using FACSAria™ Fusion Cell Sorter (Becton Dickinson).

Both samples were checked for viability and cell concentration using the Countess III cell counter (Thermo Fisher Scientific). Then, each sample containing 40.000 cells was loaded into a Rhapsody Single Cell Analysis System cartridge. Cell capture and cDNA synthesis were performed according to manufacturer’s instructions. Briefly, cells were isolated into nanowells by gravity, then lysed, and their mRNA was released and captured by beads present in the nanowells. Each bead contains a unique oligo named “cell label” to identify each individual bead. All beads present in the cartridge were collected and cDNA synthesis took place in a single reaction. At this point each cDNA was attached to its corresponding “cell label” oligo. For each sample an independent indexed library was prepared for whole transcriptome analysis following manufacturer’s instructions. The average size of the libraries was calculated using the 2100 Bioanalyzer (Agilent), and the concentration was determined using the Qubit^®^ fluorometer (Thermo Fisher Scientific). Libraries were combined and sequenced together in a paired-end run (51x75) using a NextSeq 2000 system (Illumina) and a P3 flowcell. Output files were processed with NextSeq 1000/2000 Control Software Suite v1.4.1. FastQ files for each sample were obtained using BCL Convert v3.6.3 software (Illumina).

Single-cell raw FastQ files from *ItgaxΔPtpn6* and *Ptpn^f/f^* samples were processed using BD Rhapsody pipeline v2.2 and aligned to the mm10 mouse reference genome (RhapRef_Mouse_WTA_2023-02.tar.gz), generating gene count matrices with 13,707 and 20,609 cells, respectively, prior to filtering. Data were imported into RStudio v4.0.3 and pre-processed and analyzed with Seurat v4.3.0.1. We removed cells with >12% mitochondrial content, as well as cells with fewer than 500 or more than 30,000 reads, based on library size distribution. Doublets were identified and excluded using scDblFinder v1.4.0. Cells were annotated based on canonical markers and SingleR v1.4.1. As our focus was on macrophage, dendritic cell, and monocyte lineages, non-relevant infiltrating populations were removed by subsetting clusters defined with Seurat’s FindClusters (resolution = 0.1). After filtering, the dataset comprised a total of 27,005 cells across both samples (11,358 from *Ptpn ^f/f^* and 15,647 from *ItgaxΔPtpn6*).

### 2.6 Statistical analysis

Data analysis was performed using GraphPad Prism software v.10.0 (GraphPad Software). Variables were presented as mean ± SEM, unless otherwise stated. The Shapiro–Wilk normality test was used to determine whether the samples followed a normal distribution. Comparisons between two groups were performed using two-tailed unpaired Student’s t-tests (normal distribution) or Mann–Whitney U tests (non-normal distribution). For time-course data, a repeated-measures Two-Way ANOVA test with Holm-Sidak correction was performed. Survival analysis was performed following the Log-Rank (Mantel-Cox) statistical test.

## 3 Results

### 3.1 SHP-1 depletion in CD11c ^+^ cells delays tumor rejection

**Figure 1:**
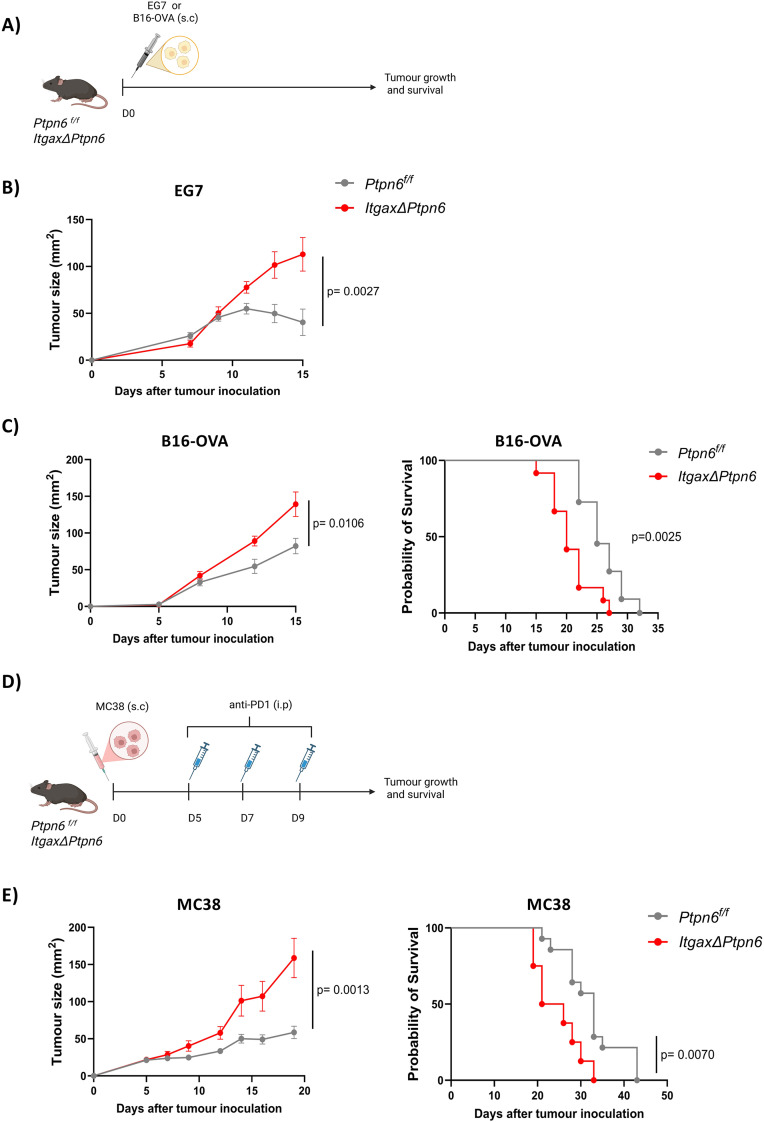
Depletion of SHP-1 in CD11c+ cells impairs rejection of different tumor cell lines. **(A)** EG7 or B16-OVA tumor cells (1x10^6^ and 5x10^5^, respectively) were inoculated subcutaneously in the right flank of *ItgaxΔPtpn6* or control *Ptpn6^f/f^* mice, and tumor growth as well as survival rate were monitored. **(B)** Tumor growth curve of EG7 tumor (n= 14 in *Ptpn6 ^f/f^* and n=10 in *ItgaxΔPtpn6* groups). **(C)** B16-OVA tumor growth (left) and survival (right) (n=11 in *Ptpn6^f/f^* and n=12 in *ItgaxΔPtpn6* groups). **(D)** MC38 tumor cells (5x10^5^) were inoculated subcutaneously in the right flank of *ItgaxΔPtpn6*, or control *Ptpn6 ^f/f^* mice and they were treated intraperitoneally with 200 µg of anti-PD1 antibody 5, 7 and 9 days after tumor inoculation. Tumor growth and survival rate were monitored. **(E)** MC38 tumor growth (left) and survival (right) (n=14 in *Ptpn6^f/f^* and n=8 in *ItgaxΔPtpn6* groups). All graphs show data pooled from two independent experiments, with biological replicates represented individually and the mean ± SEM indicated. Repeated-measured Two-Way ANOVA test with Holm-Sidak correction was performed for tumor growth, while Log-rank (Mantel-Cox) text was used in for survival analysis.

To study the role of SHP-1 in CD11c^+^ antigen-presenting cells in the context of anti-tumor immunity, we generated *ItgaxΔPtpn6* (*Itgax^Cre^Ptpn6^f/f^*) and control mice (*Ptpn6 ^f/f^*). Following injection of EG7 lymphoma in these mice (**Figure 1A**), tumor rejection was delayed in *ItgaxΔPtpn6* mice (mean tumor size of 112.9 ± 17.9 vs 40.4 ± 14.1 mm^2^ at day 15) (**Figure 1B****).** Similarly, B16-OVA melanoma rejection was delayed in *ItgaxΔPtpn6* mice (mean difference of 57 ± 19.9 mm^2^ on day 15), with a concomitant decrease in survival (**Figure 1C**).

In contrast to the EG7 and B16-OVA models, MC38 tumors are not effectively controlled in wild-type mice without checkpoint blockade, thereby limiting the ability to resolve SHP-1–dependent immune effects under basal conditions. Therefore, we used PD-1 blockade in this model to uncover functional differences in anti-tumor immunity. MC38 tumor cells were subcutaneously injected into *ItgaxΔPtpn6*, or control mice, and they were treated with anti-PD1 immunotherapy 5, 7, and 9 days after tumor inoculation (**Figure 1D**). Notably, in this immune checkpoint blockade setting, the absence of SHP-1 in CD11c^+^ cells in *ItgaxΔPtpn6* mice also resulted in impaired ability to control tumor growth compared to control mice (tumor mean size difference of 100.3 ± 27.8 mm^2^ at day 19 after tumor inoculation). Survival rates were also decreased in *ItgaxΔPtpn6* mice compared to littermate controls (median survival of 24 and 33 days in *ItgaxΔPtpn6* and *Ptpn6^f/f^* mice, respectively) (**Figure 1E**). These results unexpectedly indicate that intrinsic SHP-1 expression in CD11c^+^ cells contributes to effective tumor rejection. A limitation of the MC38 model is the requirement for PD-1 blockade to uncover immune phenotypes, which may partially confound interpretation of SHP-1–specific effects.

### 3.2 cDC1s and macrophages require SHP-1 to induce anti-tumor immunity

Our previous results showed that SHP-1 in tumor-infiltrating CD11c^+^ cells is essential for effective tumor rejection. However, since many immune cell populations within the TME express CD11c, we could not determine which specific cell types depend on SHP-1 to control tumor growth.

**Figure 2:**
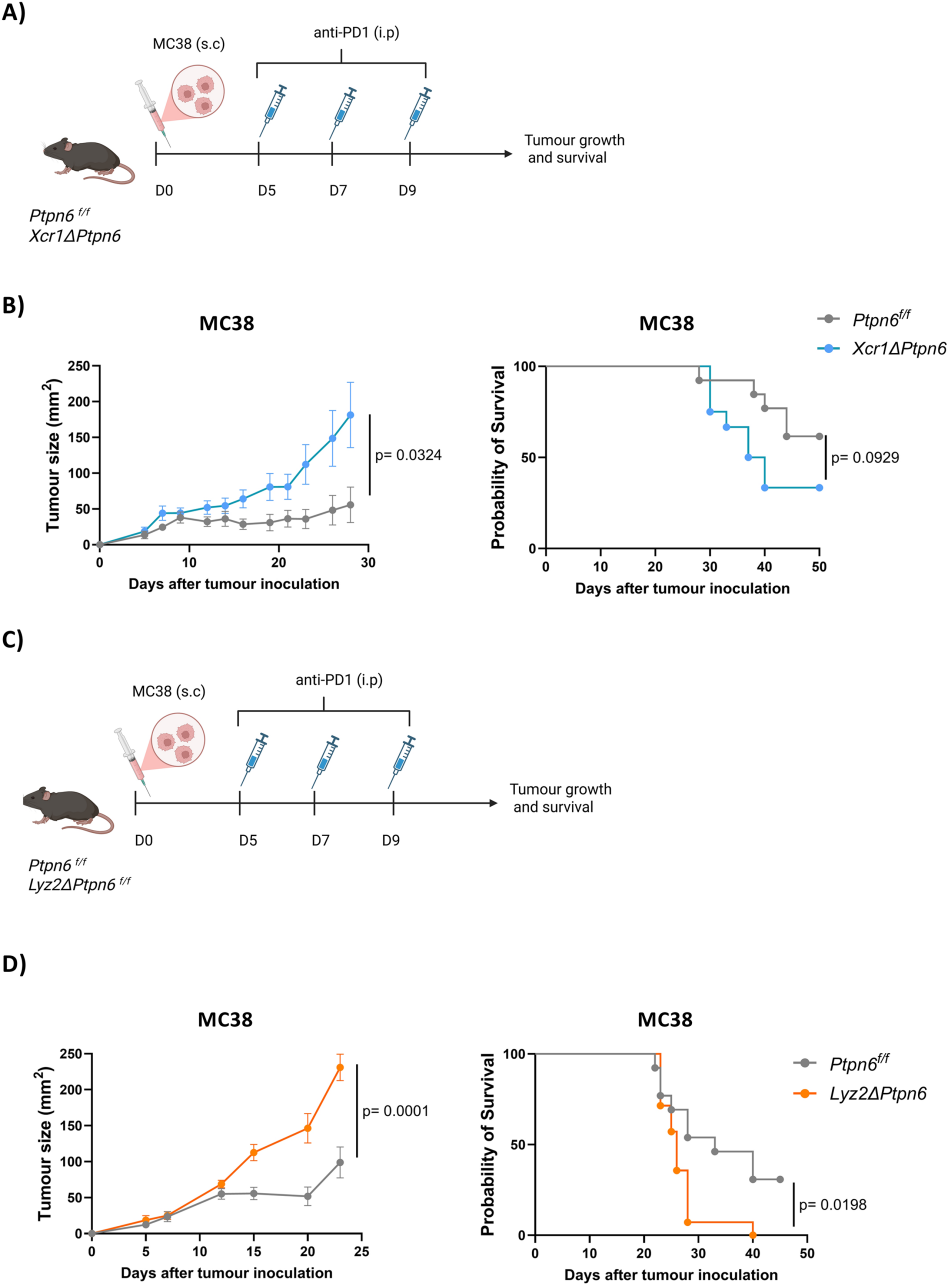
SHP-1 in cDC1s and macrophages contributes to effective anti-tumor response. **(A)** MC38 tumor cells (5x10^5^) were inoculated subcutaneously in the right flank of *Xcr1ΔPtpn6*, or control *Ptpn6^f/f^* mice and they were treated intraperitoneally with 200 µg of anti-PD1 antibody 5, 7 and 9 days after tumor inoculation. Tumor growth and survival rate were monitored. **(B)** MC38 tumor growth (left) and survival (right) (n=13 in *Ptpn6^f/f^* and n=12 in *Xcr1ΔPtpn6* groups) **(C)** MC38 tumor cells (5x10^5^) were inoculated subcutaneously in the right flank of *Lyz2ΔPtpn6* or control *Ptpn6^f/f^* mice and they were treated intraperitoneally with 200 µg of anti-PD1 antibody 5, 7 and 9 days after tumor inoculation. Tumor growth and survival rate were monitored. **(D)** MC38 tumor growth (left) and survival (right) (n=13 in *Ptpn6^f/f^* and n=14 in *Lyz2ΔPtpn6*groups). All graphs show data pooled from two independent experiments, with biological replicates represented individually and the mean ± SEM indicated. Repeated-measured Two-Way ANOVA test with Holm-Sidak correction was performed for tumor growth, while Log-rank (Mantel-Cox) text was used in for survival analysis.

Among these, conventional type 1 dendritic cells (cDC1s) express high levels of CD11c and are potent inducers of CD8^+^ T cell responses through cross-presentation, a process essential for tumor rejection. We therefore asked whether SHP-1 deficiency in cDC1s could contribute to the phenotype observed. To address this question, we generated *Xcr1*Δ*Ptpn6* (*Xcr1^Cre^Ptpn6 ^f/f^*) mice in which SHP-1 protein is specifically depleted in XCR1^+^ cDC1s. *Xcr1*Δ*Ptpn6* mice or control *Ptpn6 ^f/f^* littermates were injected with MC38 tumor and treated with anti-PD1 5, 7, and 9 days after tumor inoculation (**Figure 2A**). Similar to what we observed in *ItgaxΔPtpn6* mice, specific depletion of SHP-1 in cDC1s resulted in larger tumors (a mean tumor size difference of 125.6 ± 52 mm^2^ by day 28 after tumor inoculation) and reduced survival compared with controls (**Figure 2B**). These results demonstrate that cDC1s require SHP-1 for limiting tumor growth.

Tumor-associated macrophages (TAMs) also express CD11c and are essential to induce anti-tumor immunity, as previously described. We wondered whether SHP-1 depletion in macrophages would have a similar or distinct effect compared with cDC1s in the context of tumor rejection. To address this, we crossed the *Ptpn6 ^f/f^* mice with *Lyz2^Cre^* mice to generate *Lyz2*Δ*Ptpn6* mice (*Lyz2^Cre^ Ptpn6 ^f/f^)*. Given the high Lyz2 expression in TAMs, this model enabled us to specifically evaluate the role of SHP-1 in macrophage-mediated anti-tumor immunity. Remarkably, SHP-1 loss in Lyz2-expressing cells yielded similar results to those obtained with the *Xcr1*Δ*Ptpn6* model. Following MC38 tumor inoculation and anti-PD1 treatment (**Figure 2C**), *Lyz2*Δ*Ptpn6* mice exhibited impaired tumor rejection, showing larger tumors (mean tumor size of 230 ± 18.4 and 98.8 ± 21.5 mm^2^ at day 23 in *Lyz2*Δ*Ptpn6* and *Ptpn6 ^f/f^* mice respectively) and reduced survival compared with controls (**Figure 2D****).** These results demonstrate that SHP-1 expression in both cDC1s and macrophages contributes to tumor rejection, and that SHP-1 deficiency in either population compromises the response to MC38 tumors treated with anti-PD-1. Notwithstanding, it should be noted that Lyz2-Cre–mediated recombination is not restricted to macrophages but also occurs in neutrophils and subsets of monocytes. Therefore, genetic deletion in this model affects a broader myeloid compartment rather than macrophages exclusively.

### 3.3 scRNA-seq analysis of tumor-infiltrating CD11c ^+^ immune cells

**Figure 3:**
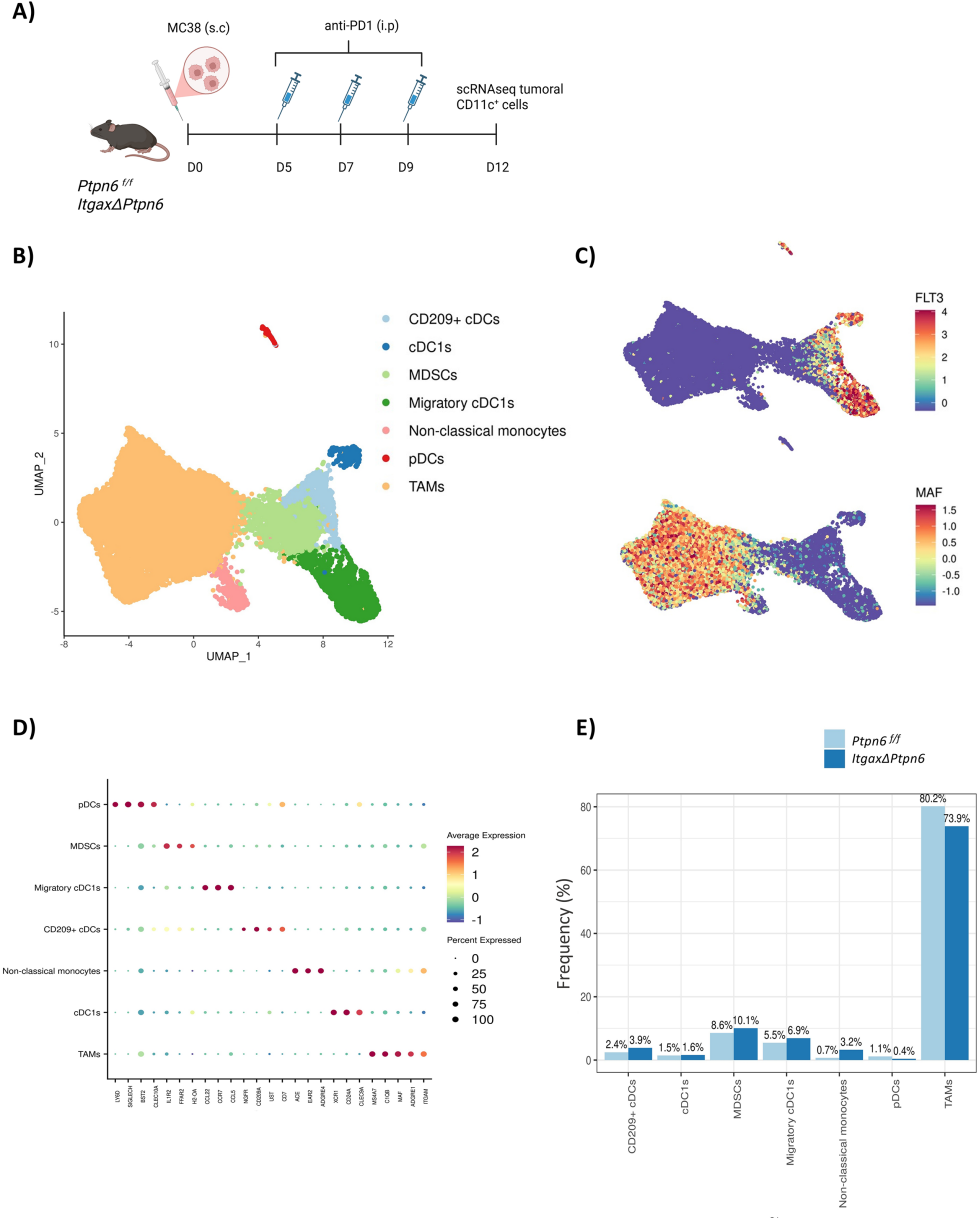
scRNA-seq of tumor infiltrating CD11c^+^ cells in *ItgaxΔPtpn6* model. **(A)** MC38 tumor cells (5x10^5^) were inoculated subcutaneously in the right flank of *ItgaxΔPtpn6* or control *Ptpn6 ^f/f^* mice and they were treated intraperitoneally with 200 µg of anti-PD1 antibody 5, 7 and 9 days after tumor inoculation. CD11c^+^ tumoral infiltrating cell were sorted on day 12 after tumor injection and scRNA-seq was performed. **(B)** Uniform Manifold Approximation and Projection (UMAP) plot of cell populations identified by scRNA-seq analysis. Each dot represents an individual cell, colored according to its assigned cell type. **(C)** Feature plots displaying expression levels of FLT3 (top) and MAF (bottom). **(D)** Dot plot of the main markers used to identify each cluster based on transcriptomic profile. **(E)** Percentage of every cluster identified in both samples.

Given the critical role of SHP-1 in CD11c^+^ tumor-infiltrating cells, particularly cDC1s and macrophages, we next performed a scRNA-seq of tumor-infiltrating CD11c^+^ cells to profile their transcriptional changes upon SHP-1 loss. To this aim, *ItgaxΔPtpn6* and control mice were injected subcutaneously with MC38 tumor cells and treated with anti-PD1 on days 5, 7 and 9 after tumor inoculation. Tumors were harvested on day 12 (**Figure 3A**), corresponding to the time point when tumor growth began to diverge between genotypes (**Figure 1E**).

For each condition, CD11c^+^ cells infiltrating tumors from five pooled mice were sorted and sequenced for scRNA-seq. As a control for deletion specificity, scRNA-seq feature plots confirmed efficient loss of Ptpn6 expression in *ItgaxΔPtpn6* cells compared with *Ptpn6 ^f/f^* controls (**Supplementary Figure S1A**). In contrast, Ptpn11 (SHP2) expression was comparable across genotypes (**Supplementary Figure S1B**), indicating that the observed transcriptional changes are not attributable to compensatory alteration in SHP2 mRNA expression. A total of 15.647 CD11c^+^ cells from *ItgaxΔPtpn6* mice and 11.358 CD11c^+^ cells from *Ptpn6 ^f/f^* control littermates were sequenced, identifying seven clusters (**Figure 3B****).** Based on the expression of FLT3 and MAF, we could easily differentiate between DCs and macrophages, respectively, although some cells did not express either marker (**Figure 3C**). Cluster annotation was performed using specific gene signatures (**Figure 3D**; **Supplementary Figure S2A**): TAMs (*Ms4a7, C1qb, Maf, Adgre1, Itgam*), plasmacytoid dendritic cells (pDCs) (*Ly6d, SiglecH, Bst2, Clec10A*), non-classical monocytes (*Ace, Ear2, Adgre4*), cDC1s (*Xcr1, CD24A, Clec9A*), migratory cDCs (*Ccr7, Ccl5, Ccl22*), CD209^+^ cDCs (*CD209A, Ngfr, CD7, Ust*), myeloid-derived suppressor cells (MDSCs) (*Ffar2, H2-OA, Il1r2*). Notably, the migratory cDC cluster showed high IRF8 expression (**Supplementary Figure S2B**), a key transcription factor for cDC1 differentiation from pre-cDCs (1), suggesting that these CCR7^+^ cDCs may derive from immature cDC1s. During activation, cDC1s likely downregulate *Xcr1* (**Supplementary Figure S2B**) and upregulate *Ccr7*, consistent with previous reports (32). Quantitative analysis revealed an increase in non-classical monocytes in *ItgaxΔPtpn6* tumors compared with controls, along with modest increases in migratory cDCs, CD209^+^ cDCs, and MDSCs. In contrast, pDCs and TAMs were slightly reduced (**Figure 3E**).

To determine whether SHP-1 deletion in CD11c^+^ cells alters the abundance of major myeloid populations in the tumor microenvironment or tdLN, we performed flow cytometry analysis at day 12 following the experiment setting described in **Figure 3A**. Quantification of cDCs, TAMs, monocytes, and neutrophils revealed no significant differences between Ptpn6^f/f^ and ItgaxΔPtpn6 mice in either tumors or tdLN (**Supplementary Figures S3A, B**). Notably, we observed a trend toward fewer CD11c^+^ and CD11c^-^ TAMs in tumors from ItgaxΔPtpn6 mice (**Supplementary Figure S3B**).

### 3.4 SHP-1 loss impairs anti-tumor immunity in tumor-associated macrophages

**Figure 4:**
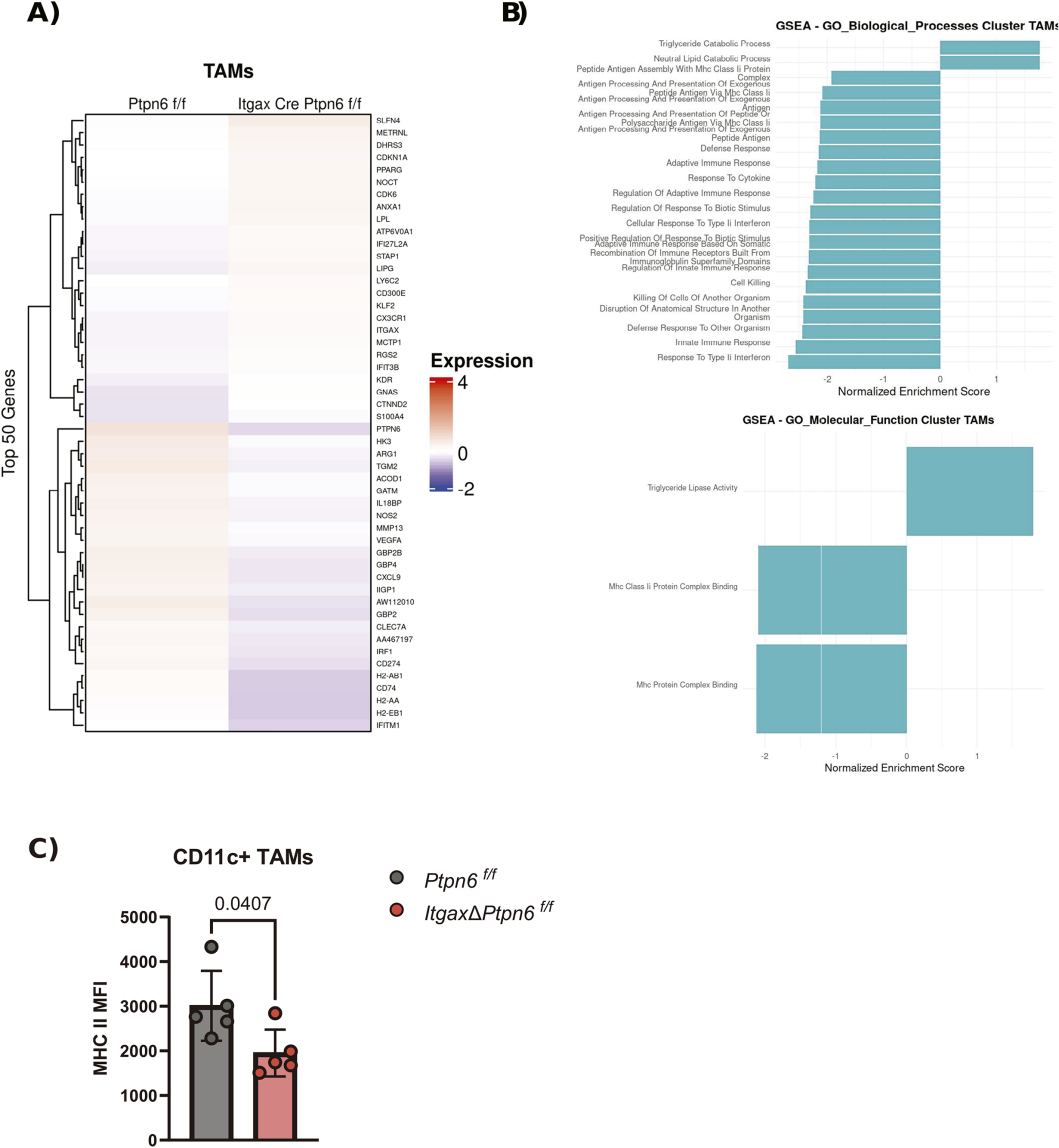
SHP-1 loss impairs anti-tumor immunity in tumor associated macrophages. **(A)** Heatmap showing the top 50 differentially expressed genes within the tumor-associated macrophages (TAMs) cluster identified by scRNA-seq from *ItgaxΔPtpn6* or control *Ptpn6 ^f/f^* mice injected with MC38 tumoral cells and treated with anti-PD1. **(B)** Gene Set Enrichment Analysis (GSEA) performed from the TAMs cluster comparing *ItgaxΔPtpn6* vs *Ptpn6 ^f/f^* (Gene ontology biological processes is shown in the top, while gene ontology molecular function is represented at bottom). Positive normalized enrichment score means an upregulated pathway in the ItgaxΔPtpn6. **(C)** Flow cytometry analysis of MHC II mean fluorescence intensity (MFI) in CD11c^+^ tumor-associated macrophages isolated from tumors harvested on day 12 after subcutaneous inoculation of MC38 cells (5 × 10^5^) in *ItgaxΔPtpn6* or *Ptpn6 ^f/f^* mice treated with 200 µg of anti-PD1 antibody 5, 7 and 9 days after tumor inoculation. Data shown in **(C)** is representative of two independent experiments, with biological replicates represented individually and the mean ± SEM indicated. Unpaired t-test analysis was performed.

TAMs are a major component of TME, where they regulate immune responses that can either promote tumor elimination or support tumor progression. Transcriptomic analysis of TAMs from our scRNA-seq (**Figure 4A**) showed that SHP-1 loss led to downregulation of pathways required for effective anti-tumor immunity, including type II interferon response, cell killing, and innate immune activation (**Figure 4B**). This shift is consistent with polarization toward an anti-inflammatory phenotype, which could explain the impaired tumor rejection observed in *ItgaxΔPtpn6* mice.

Consistent with this, TAMs from tumors in *ItgaxΔPtpn6* mice upregulated lipid metabolism pathways compared with *Ptpn6 ^f/f^* controls, particularly those linked to triglyceride and neutral lipid catabolism (**Figure 4B**). Enhanced lipid catabolism in TAMs is a hallmark of anti-inflammatory metabolic reprogramming, reducing the ability to present antigen and impairing anti-tumor T cell responses (33–35). In line with this, we also observed downregulation of MHC assembly and antigen presentation pathways (**Figure 4B**), which are essential for T cell activation via tumor antigen presentation. Importantly, these transcriptomic changes were supported by flow cytometry analysis, which revealed reduced MHC II levels in CD11c^+^ TAMs from *ItgaxΔPtpn6* mice compared with *Ptpn6^f/f^* controls (**Figure 4C**). Non-classical monocytes exhibited similar features, including a downregulation of interferon gamma response (**Supplementary Figure S4A**), while MDSCs and other clusters showed a similar trend (**Supplementary Figure S4B****).** These results indicate that SHP-1 deficiency disrupts interferon signaling and MHC complex formation in multiple myeloid cells, thereby impairing their ability to mount an effective anti-tumor response.

### 3.5 SHP-1 deficiency reshapes activation and interferon-responsive programs in cDC1s

**Figure 5:**
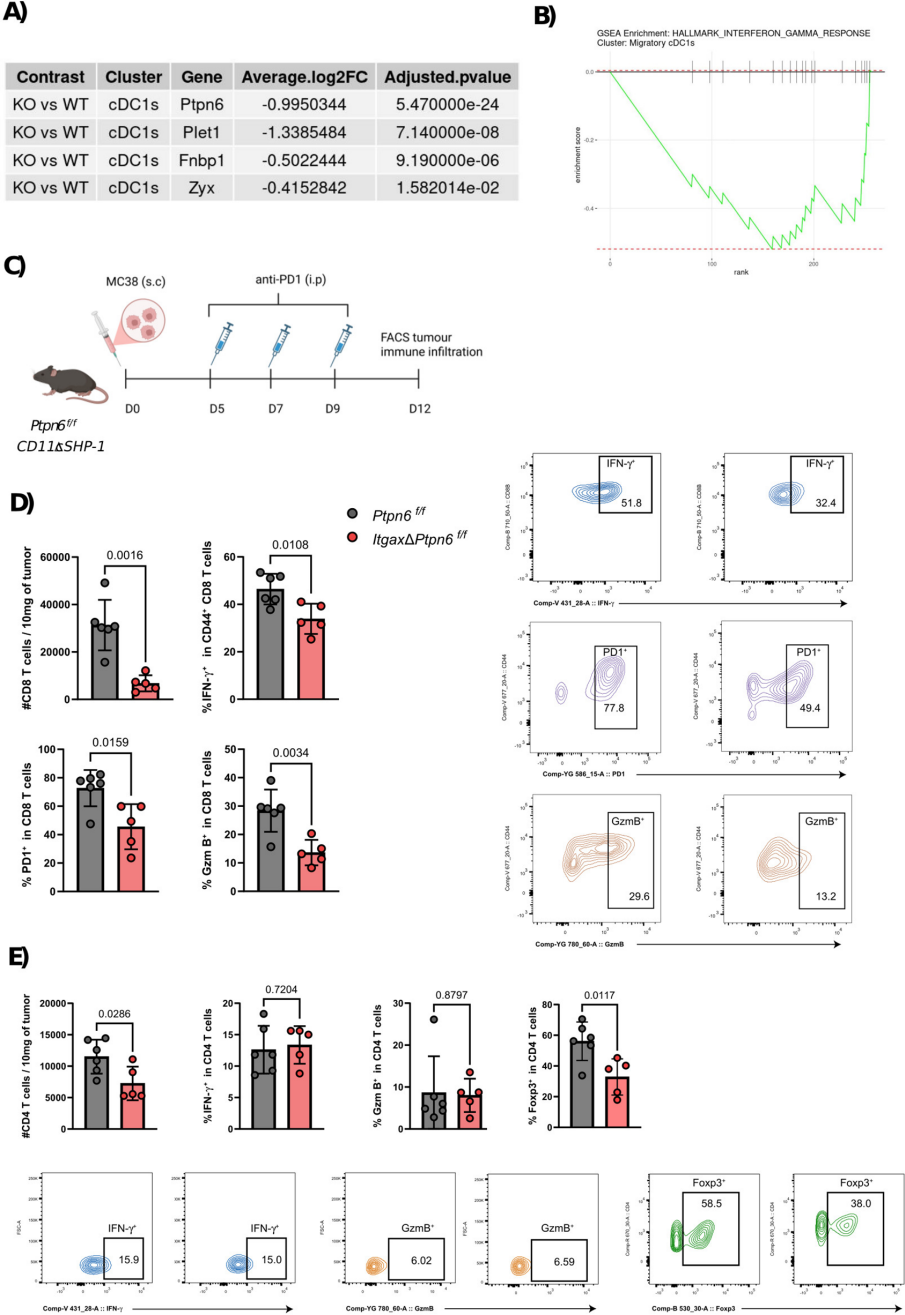
SHP-1 regulates migratory and activation programs in cDC1s. **(A)** Table showing selected differentially expressed genes in XCR1^+^ cDC1 between KO (*ItgaxΔPtpn6*) and WT (*Ptpn6^f/f^*). Negative Average log2 fold change means a downregulated gene in ItgaxΔPtpn6 compared to control. **(B)** GSEA plot showing a decreased enrichment of IFN-γ response pathway in migratory cDC1s from *ItgaxΔPtpn6* compared to control. **(C)** MC38 tumor cells (5x10^5^) were inoculated subcutaneously in the right flank of *ItgaxΔPtpn6* or control *Ptpn6^f/f^* mice, and they were treated intraperitoneally with 200 µg of anti-PD1 antibody 5, 7, and 9 days after tumor inoculation. On day 12, tumor immune cell infiltration was analyzed by flow cytometry (FACS). **(D)** Flow cytometry analysis of tumor-infiltrating CD8^+^ T cells in *ItgaxΔPtpn6* or control *Ptpn6^f/f^* mice. Representative dot plots are shown (right). Quantification (left to right) includes: absolute number of CD8^+^ T cells per 10 mg of tumor tissue, percentage of IFN-γ^+^ cells among CD44^+^ CD8^+^ T cells, percentage of PD-1^+^ cells among CD8^+^ T cells, and percentage of granzyme B^+^ CD8^+^ T cells. **(E)** Flow cytometry analysis of tumor-infiltrating CD4^+^ T cells from ItgaxΔPtpn6 and control Ptpn6^f/f^ mice. Representative dot plots are shown (bottom). Quantification (left to right) includes: absolute number of CD4^+^ T cells per 10 mg of tumor tissue, percentage of IFN-γ^+^ cells among CD4^+^ T cells, percentage of granzyme B^+^ CD4^+^ T cells, and percentage of Foxp3^+^ regulatory T cells among CD4^+^ T cells. Each dot represents an individual mouse; bars indicate mean ± SEM. The data are representative of two independent experiments. Statistical significance was determined using an unpaired t-test.

cDC1s uptake tumor-associated antigens and migrate to the TDLNs, where they prime naïve T cells. This process requires upregulation of CCR7 to direct migration of cDC1s from the tumor to the dLNs. In *ItgaxΔPtpn6* mice, tumoral cDC1s showed downregulation of *Plet1* gene, whose absence impairs DC migratory capacity (36), as well as reduced expression of *Fnbp1* and *Zyx*, genes critical for cytoskeletal dynamics and actin polymerization (37) (**Figure 5A****).** These results are consistent with SHP-1’s role in coordinating neutrophil and DC migration through integrin signaling (38).

Furthermore, transcriptomic profiling revealed downregulation of IFN-γ response pathways in migratory CCR7^+^ cDC1s (**Supplementary Figure S4C**; **Figure 5B**), which are crucial for cDC1 maturation, survival, and cross-priming activity. To complement these transcriptomic findings, we assessed the surface expression of key antigen-presentation and co-stimulatory molecules on DC subsets in tumors and tdLNs. Flow cytometry analysis of CD80, CD86, and MHC II revealed subset- and compartment-dependent differences between genotypes (**Supplementary Figures S5A, B**), without a consistent pattern indicative of a uniform defect in DC activation due to SHP-1 deficiency. Migratory cDC1s in tdLNs did not show major differences in these markers, whereas resident cDC1s displayed a mixed phenotype characterized by reduced CD80 and increased CD86. Thus, although the scRNA-seq data suggest altered migratory and IFN-responsive programs, the flow cytometry data do not directly confirm a phenotypic defect in migratory cDC1s at the protein level. These results suggest that SHP-1 deletion alters DC activation programs in a context-dependent manner rather than globally suppressing DC maturation.

We next examined the downstream consequences of SHP-1 loss in CD11c^+^ cells on intratumoral T cell responses. We analyzed immune infiltration by tumoral CD8^+^ T cells following SHP-1 depletion in CD11c^+^ cells. Flow cytometry at day 12 after MC38 inoculation (**Figure 5C**) showed a 4.6-fold reduction in intratumoral CD8^+^ T cell infiltration in *ItgaxΔPtpn6* compared with control mice, along with decreased PD1 expression. Effector CD8^+^ T cells also produced less IFN-γ (33.9 ± 2.84% vs 46.4 ± 2.6% in controls) (**Figure 5D**). In addition, intratumoral CD8^+^ T cells from *ItgaxΔPtpn6* mice expressed lower levels of granzyme B, consistent with impaired cytotoxic effector potential (**Figure 5E**).

Notably, we also observed a reduction in the absolute number of tumor-infiltrating CD4^+^ T cells in *ItgaxΔPtpn6 mice* (**Figure 5E**). However, the frequencies of IFN-γ^+^ and granzyme B^+^ cells among tumor CD4^+^ T cells were comparable across genotypes (**Figure 5E**). Conversely, we observed a reduced Treg frequency in tumors from *ItgaxΔPtpn6* mice, which may reflect impaired recruitment or local expansion of these cells within the tumor microenvironment, potentially secondary to defective myeloid cell function.

These data indicate that SHP-1 deletion in CD11c^+^ cells is associated with reduced T-cell accumulation within tumors and impaired CD8^+^ T-cell effector responses, consistent with altered myeloid cell function in the tumor microenvironment.

To determine whether T cell alterations were also observed in the tumor-draining lymph node, we analyzed tdLNs from the same experimental setting by flow cytometry. No significant differences were detected in the absolute numbers of CD4^+^ or CD8^+^ T cells, nor in the frequency of PD-1^+^ CD8^+^ T cells, between ItgaxΔPtpn6 and Ptpn6 f/f mice (**Supplementary Figures S7A, B**). Intriguingly, tdLN CD8^+^ T cells from *ItgaxΔPtpn6* mice showed an increased frequency of PD1^+^ and IFN-γ–producing cells compared with controls (**Supplementary Figure S7A**), while the frequency of Foxp3^+^ Treg cells was also increased, suggesting a compensatory regulatory response.

This discrepancy likely reflects compartment-specific dynamics, with differential effects of SHP-1 deficiency in CD11c^+^ cells on T cell priming in the tdLN versus their accumulation or maintenance within the tumor.

## 4 Discussion

The TME comprises a diverse array of cellular components that collectively influence tumor progression and therapeutic response. Both innate and adaptive immune cells within the TME play critical roles in suppressing tumor growth, and immune checkpoint blockade enhances the capacity of the immune system to attack cancer by targeting regulatory pathways such as PD-1, PD-L1, and CTLA-4 (39). As a result, several immune checkpoint inhibitors (ICIs), including nivolumab, pembrolizumab, atezolizumab, and ipilimumab, have been approved in the last years, significantly improving survival and response rates in cancers such as lung cancer, melanoma, and renal cell carcinoma (40).

SHP-1, a protein tyrosine phosphatase broadly expressed in hematopoietic cells, is a key regulator of multiple immune signaling pathways and has emerged as a promising target for modulating immune responses in both cancer and autoimmune disease (19, 41, 42). Inhibition of SHP-1 activity offers an attractive immunotherapeutic strategy for cancer treatment. However, our results using the *ItgaxΔPtpn6* model showed that SHP-1 deletion in tumor-infiltrating myeloid cells alters the transcriptional and functional programs of both TAMs and cDC1s. Unexpectedly, whereas SHP-1 loss in T lymphocytes has been shown to enhance proliferation, cytotoxicity, and tumor rejection (25, 26), deletion of SHP-1 in myeloid cells instead impaired anti-tumor immunity. This striking contrast underscores the cell-type–specific roles of SHP-1 in immune regulation. In TAMs, SHP-1 loss promotes a lipid-metabolic phenotype accompanied by reduced interferon signaling and antigen presentation transcriptional programs, which we validated by observing reduced MHC-II expression at the protein level. In contrast, cDC1s displayed alterations in interferon-responsive and migratory transcriptional programs rather than a uniform reduction in antigen-presentation or co-stimulatory markers. These alterations were associated with reduced CD8^+^ T cell infiltration and diminished effector function. Thus, while SHP-1 inhibition may benefit lymphocyte-driven immunity, our findings reveal that SHP-1 is essential for preserving myeloid cell function within the TME, cautioning against its indiscriminate targeting in cancer immunotherapy.

Because CD11c is expressed by multiple myeloid cell populations, including dendritic cells, macrophages, and subsets of monocytes, the impaired tumor rejection in *ItgaxΔPtpn6* mice likely reflects combined effects across these subsets. Our results with *Xcr1*Δ*Ptpn6* mice demonstrate that cDC1s require SHP-1 to restrain tumor progression, while the *Lyz2*Δ*Ptpn6* revealed a similar SHP-1 requirement for anti-tumor immunity by macrophage populations. Although *Lyz2*-Cre is not strictly macrophage-specific and is also active in neutrophils and monocytes (31), the convergence of phenotypes across *Xcr1*- and *Lyz2*-driven deletions strongly supports that both cDC1s and TAMs rely on SHP-1 for anti-tumor immunity. These evidences highlight that SHP-1 depletion in either cDC1s or macrophages compromises anti-tumor immunity. Importantly, because Lyz2-Cre can also target neutrophils, we cannot exclude a contribution from this population to the observed phenotype, which represents a limitation of the current study. Previous work using *Cx3cr1*-Cre showed that SHP-1-deficient macrophages exhibit enhanced phagocytic activity *in vitro* (20). Notably, that study explicitly reported that *Itgax*-Cre and *Mrp8*-Cre deletions led to severe baseline inflammation, which precluded their analysis of tumor models in a myeloid-specific mouse model. As a result, their *in vivo* tumor experiments relied on a global, inducible deletion that triggered systemic inflammation and multi-compartment immune activation, complicating the attribution of effects to specific myeloid subsets. In contrast, in our model, SHP-1 deletion using *Itgax*-Cre– and *Lyz2*-Cre did not cause overt inflammation at the time of tumor challenge, enabling direct assessment of the cell-intrinsic role of SHP-1 in cDC1s and macrophage-enriched populations within the TME.

To investigate the mechanisms underlying the impaired anti-tumor response in our *ItgaxΔPtpn6* model, we performed scRNA-seq on tumor-infiltrating CD11c^+^ cells. This approach allowed us to characterize the myeloid landscape in the tumor and revealed distinct populations, including cDC1s, migratory CCR7^+^ cDC1s, TAMs, and additional subsets. TAMs from *ItgaxΔPtpn6* mice adopted a more anti-inflammatory phenotype, with downregulation of pathways involved in cell killing, interferon signaling, and innate immune activation, as well as impaired MHC class I antigen presentation. Importantly, these transcriptional changes were supported by protein-level validation, as TAMs from SHP-1–deficient tumors displayed reduced MHC-II expression in both CD11c^-^ and CD11c^+^ subsets. These changes suggest that SHP-1 deficiency compromises TAM-mediated tumor control. Similarly, cDC1s from *ItgaxΔPtpn6* mice exhibited transcriptional alterations in pathways associated with migration and interferon-γ responsiveness, suggesting that SHP-1 deficiency may impair the ability of these cells to coordinate effective T-cell immunity. Consistent with this, flow cytometry revealed reduced CD8^+^ T cell infiltration, lower PD-1 expression, and decreased IFN-γ production in tumors from *ItgaxΔPtpn6* mice. We also observed reduced numbers of tumor-infiltrating CD4^+^ T cells, although their effector cytokine production remained largely unchanged. SHP-1 is part of a regulatory feedback loop controlling IFN-γ/STAT1 signaling, as interferon signaling induces SHP-1 expression through STAT1-dependent mechanisms (43, 44). Disruption of this feedback upon SHP-1 deletion may contribute to the altered interferon-responsive transcriptional programs observed in cDC1s.

Although our data identify defects in cDC1 migratory and IFN-γ–responsive programs *in vivo*, these transcriptional alterations were not matched by a clear phenotypic change in migratory cDC1s in tumor-draining lymph nodes, as flow cytometry did not reveal major differences in CD80, CD86, or MHC II expression in this subset. This discrepancy suggests that the scRNA-seq signatures may reflect altered functional programs not fully captured by the protein markers analyzed here and therefore supports a more cautious interpretation of SHP-1-dependent effects on cDC1 migration. Moreover, we did not directly assess cross-presentation capacity or IFN-γ responsiveness in purified cDC1s *in vitro*. Such assays would provide important mechanistic insight into the cell-intrinsic role of SHP-1. However, these experiments were limited by the low yield of tumor-infiltrating cDC1s at the experimental endpoint. Future studies incorporating *in vitro* cross-presentation and cytokine-stimulation assays will be important for further dissecting how SHP-1 regulates cDC1 function in the tumor microenvironment.

Previous studies have shown that SHP-1 deficiency enhances DC immunogenicity in other contexts (45, 46). For example, DC-specific SHP-1 deletion was reported to cause splenomegaly, expansion of CD11c^+^ DCs, increased CD86 and CCR7 expression, and heightened proinflammatory cytokine production, resulting in augmented Th1 polarization and IFN-γ production by antigen-specific T cells (45). During infections such as *Leishmania* or viral challenge, SHP-1-deficient DCs displayed improved cross-presentation and stronger CD8^+^ T cell responses, linked to reduced endosomal acidification that enhances antigen processing and MHC class I presentation (46). Together, these findings demonstrate that SHP-1 restricts DC immunogenicity in a context-dependent manner. Our results suggest that within the tumor microenvironment, SHP-1 loss may instead lead to dysregulated dendritic cell maturation, in which phenotypic activation markers may be maintained or even increased, while the coordinated programs required for effective migration and T-cell priming are disrupted. This highlights that the TME uniquely shapes these effects, and that findings from systemic or infection models cannot be directly extrapolated to cancer.

Several SHP-1 inhibitors have been developed, including NSC-87877, sodium stibogluconate (SSG), tyrosine phosphatase inhibitor 1 (TPI-1), and suramin (47–51). However, most have shown limited efficacy in experimental preclinical tumor models (27, 28). For instance, SSG, which is approved for the treatment of leishmaniasis, entered phase I trials in patients with malignant melanoma, but failed to demonstrate meaningful benefit. More recently, allosteric inhibitors such as SB8091 have shown improved pharmacological profiles in preclinical studies, particularly in combination with anti-PD1 therapy. However, most current drug−development programs targeting “SHP” phosphatases focus on SHP−2 rather than SHP−1 (52, 53), and no selective SHP−1 inhibitors have advanced to Phase II trials (27, 28, 54). A major limitation of current compounds is their lack of SHP-1 specificity, as many targets multiple phosphatases, raising the risk of off-target effects and diminishing therapeutic efficacy (54, 55). These challenges underscore the need for developing more selective SHP-1 modulators—whether inhibitors or activators—to precisely interrogate its biological functions and fully evaluate its therapeutic potential.

Overall, our findings reveal striking cell-type–specific effects of SHP-1 in the TME and underscore the complexity of its role in regulating anti-tumor immunity. While SHP-1 inhibition may enhance lymphocyte-mediated responses, our results demonstrate that SHP-1 is required to maintain functional antigen-presentation programs in tumor-associated myeloid cells. Global SHP-1 inhibition could lead to opposing outcomes across immune compartments, with detrimental consequences. Thus, future therapeutic strategies may benefit from agents that provide cell-type–selective, transient, and reversible modulation of SHP-1 activity. A deeper understanding of how SHP-1 loss or modulation affects individual immune compartments will be critical for guiding the rational design of next-generation immunotherapies.

## Data Availability

The data presented in this study have been deposited in the ArrayExpress repository under accession number E-MTAB-16912 (“scRNA-seq of tumor infiltrating CD11c+ cells in ItgaxΔPtpn6 model”).

## Supplementary material

Supplementary Figure S1 Ptpn6 deletion does not affect Ptpn11 (SHP-2) mRNA expression in tumor-infiltrating myeloid cells. **(A)** Feature plots showing *Ptpn6* expression across tumor-infiltrating CD11c^+^ myeloid cells identified by scRNA-seq from *Ptpn6^f/f^* and *ItgaxΔPtpn6* mice bearing MC38 tumors. Expression levels are displayed on a UMAP projection, with colors indicating normalized transcript abundance. **(B)** Feature plots showing *Ptpn11* (SHP-2) expression across the same cell populations. Each point represents a single cell.

Supplementary Figure S2 Transcriptome profile of CD11c^+^ tumor-infiltrating cells by scRNA-seq. **(A)** Heatmap showing the 15 top markers for each cluster based on logFC. **(B)** Feature plots displaying expression levels of IRF8 (left) and XCR1 (right).

Supplementary Figure S3 Quantification of myeloid populations in tumor and draining lymph node. **(A)** Representative flow cytometry gating strategy used to identify major myeloid populations in MC38 tumor samples. Sequential gating identifies cDC1s, cDC2s, CD11c^+^ tumor-associated macrophages (TAMs), CD11c^-^ TAMs, monocytes, and neutrophils. **(B)** Absolute numbers of cDC1s, cDC2s, CD11c^+^ TAMs, CD11c^-^ TAMs, monocytes, and neutrophils quantified by flow cytometry in tumors (top) and tumor-draining lymph nodes (bottom) from *ItgaxΔPtpn6* and *Ptpn6^f/^*^f^ mice. MC38 tumor cells (5×10^5^) were inoculated subcutaneously, and mice were treated with anti-PD1 on days 5, 7, and 9; tissues were harvested on day 12. Each dot represents an individual mouse, and bars indicate mean ± SEM. Data are pooled from two independent experiments. Statistical significance was assessed using multiple unpaired *t*-tests.

Supplementary Figure S4 SHP-1 deficiency differentially affects the transcriptional programs of distinct tumor-infiltrating myeloid populations. **(A)** GSEA plot showing a decreased enrichment of IFN-γ response pathway in non-classical monocytes from ItgaxΔPtpn6 compared to control. **(B)** Summary table of normalized enrichment scores (NES) and adjusted p-values in MDSCs cluster. Negative NES means a downregulated pathway in ItgaxΔPtpn6 compared to control. **(C)** Heatmap showing the top 50 differentially expressed genes within the migratory cDC1s cluster identified by scRNA-seq from *ItgaxΔPtpn6* or control *Ptpn6 ^f/f^* mice injected with MC38 tumoral cells and treated with anti-PD1.

Supplementary Figure S5 Phenotypic characterization of cDC subsets in tumor and draining lymph node. MC38 tumor cells (5×10^5^) were inoculated subcutaneously into *ItgaxΔPtpn6* or control *Ptpn6 ^f/f^* mice and animals were treated with anti-PD1 on days 5, 7 and 9. Tumors and tumor-draining lymph nodes (tdLNs) were harvested on day 12 and analyzed by flow cytometry. **(A)** Mean fluorescence intensity (MFI) of CD80, CD86 and MHC II expression in tumor-infiltrating cDC1s and cDC2s. **(B)** MFI of CD80, CD86, and MHC II expression in migratory and resident cDC1s and cDC2s in tdLNs. Each dot represents an individual mouse, and bars indicate mean ± SEM. Data are pooled from two independent experiments. Statistical significance was assessed using an unpaired *t*-test.

Supplementary Figure S6 Flow cytometry gating strategy for the analysis of tumor-infiltrating CD8^+^ and CD4^+^ T cells. Representative flow cytometry gating strategy used to quantify T cell populations and effector markers in MC38 tumors, corresponding to the analyses shown in **Figures 5D, E**. Cells were first gated based on forward and side scatter (FSC-A vs SSC-A) to exclude debris, followed by singlet discrimination (FSC-A vs FSC-H) and selection of live cells using viability dye exclusion. Immune cells were then identified as CD45^+^, and CD45^+^CD90.1^+^ lymphocytes were gated based on FSC/SSC characteristics. CD3^+^ T cells were further separated into CD8^+^ and CD4^+^ subsets. Within CD8^+^ T cells, the frequencies of PD-1^+^ cells, granzyme B–producing cells, and IFN-γ–producing cells were quantified. CD44 expression was used to define activated CD8^+^ T cells for intracellular cytokine analysis. Within CD4^+^ T cells, granzyme B and IFN-γ expression were also assessed. Percentages shown represent the frequency of gated populations within the indicated parent population. Representative plots are shown.

Supplementary Figure S7 T cell phenotype in tumor-draining lymph nodes under MC38 anti–PD-1 treatment. MC38 tumor cells (5 × 10^5^) were inoculated subcutaneously into *ItgaxΔPtpn6* or control *Ptpn6 ^f/f^* mice. Mice were treated intraperitoneally with anti–PD-1 antibody on days 5, 7, and 9 after tumor inoculation. Tumor-draining lymph nodes (tdLNs) were harvested on day 12 and analyzed by flow cytometry. **(A)** Analysis of CD8^+^ T cells in tdLNs, including absolute CD8^+^ T cell numbers per lymph node, frequency of IFN-γ^+^ cells among CD44^+^ CD8^+^ T cells, and frequency of PD-1^+^ cells within CD8^+^ T cells. **(B)** Analysis of CD4^+^ T cells in tdLNs, including absolute CD4^+^ T cell numbers per lymph node and frequency of Foxp3^+^ regulatory T cells within the CD4^+^ T cell compartment. Each dot represents an individual mouse; bars indicate mean ± SEM. Data are representative of two independent experiments. Statistical significance was determined using an unpaired t-test.
