# Supplementary figures and images for: Loss of SHP-1 in CD11c+ cells impairs anti-tumor immunity
Source: Front Immunol. 2026 Apr 21;17:1710547. doi: 10.3389/fimmu.2026.1710547 (PMC13139866; doi:10.3389/fimmu.2026.1710547)

Figure S1

A)

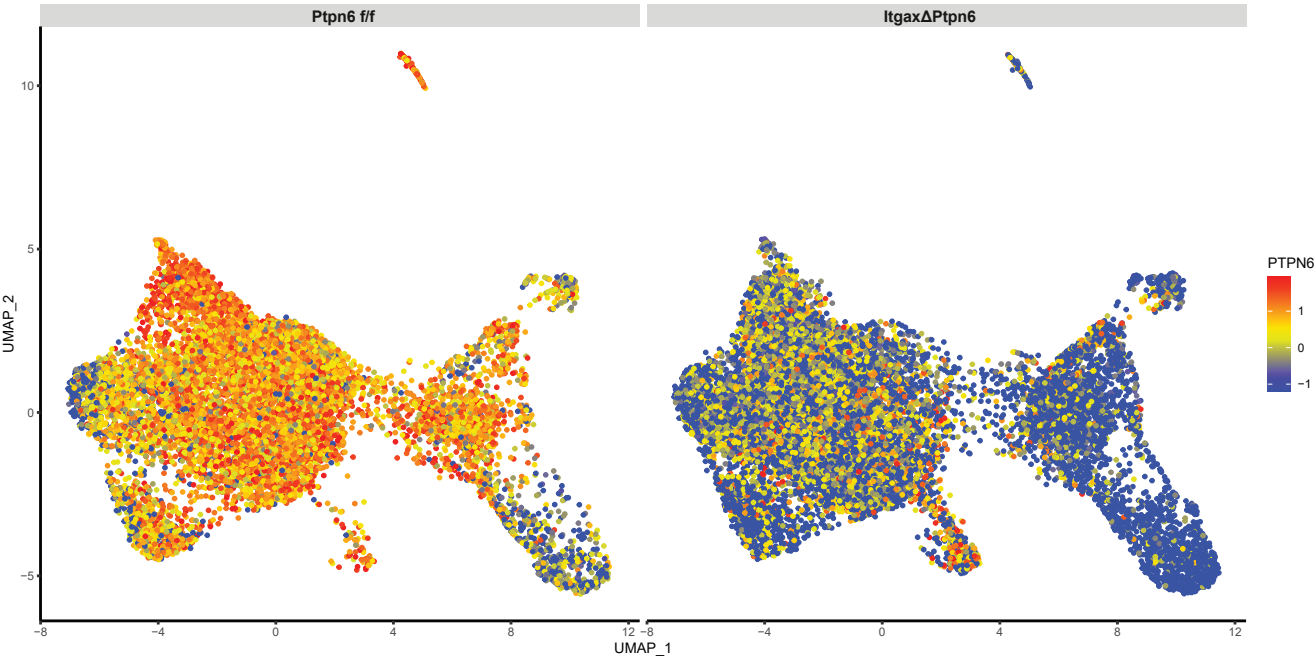

B)

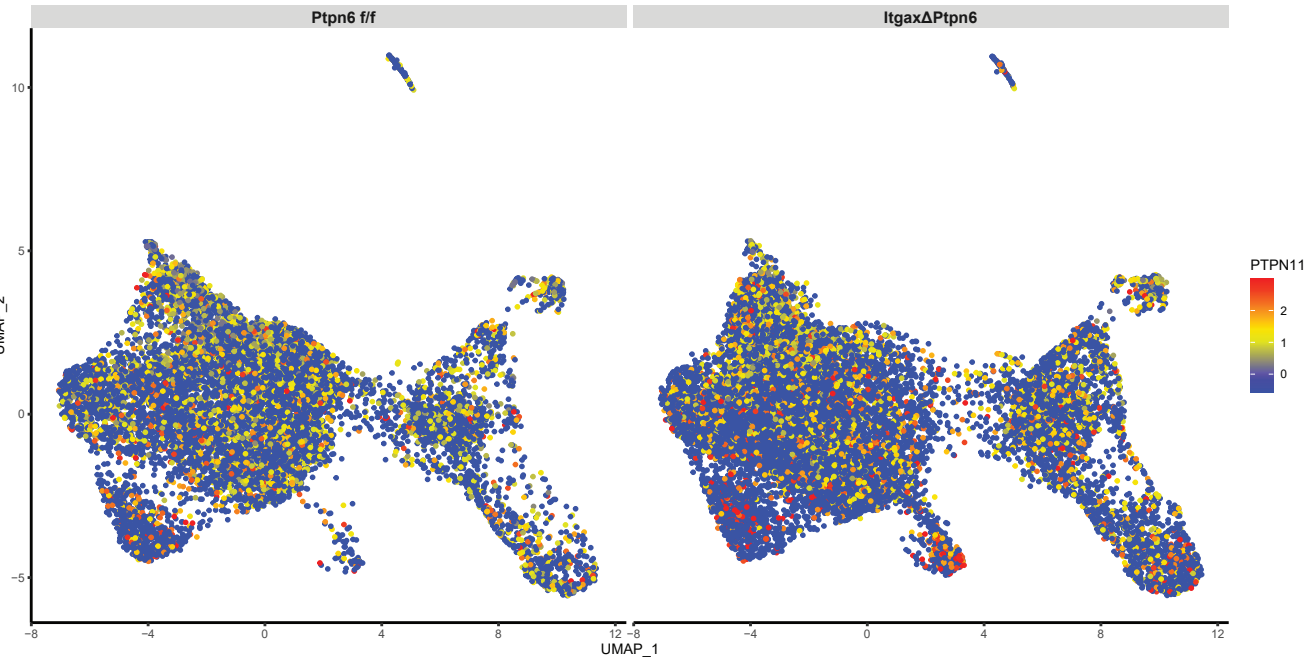

**A)**

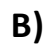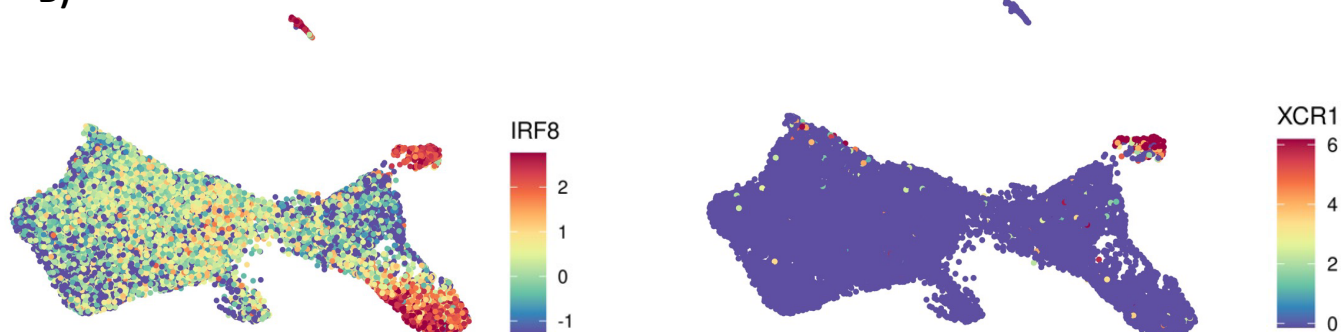

Figure S3

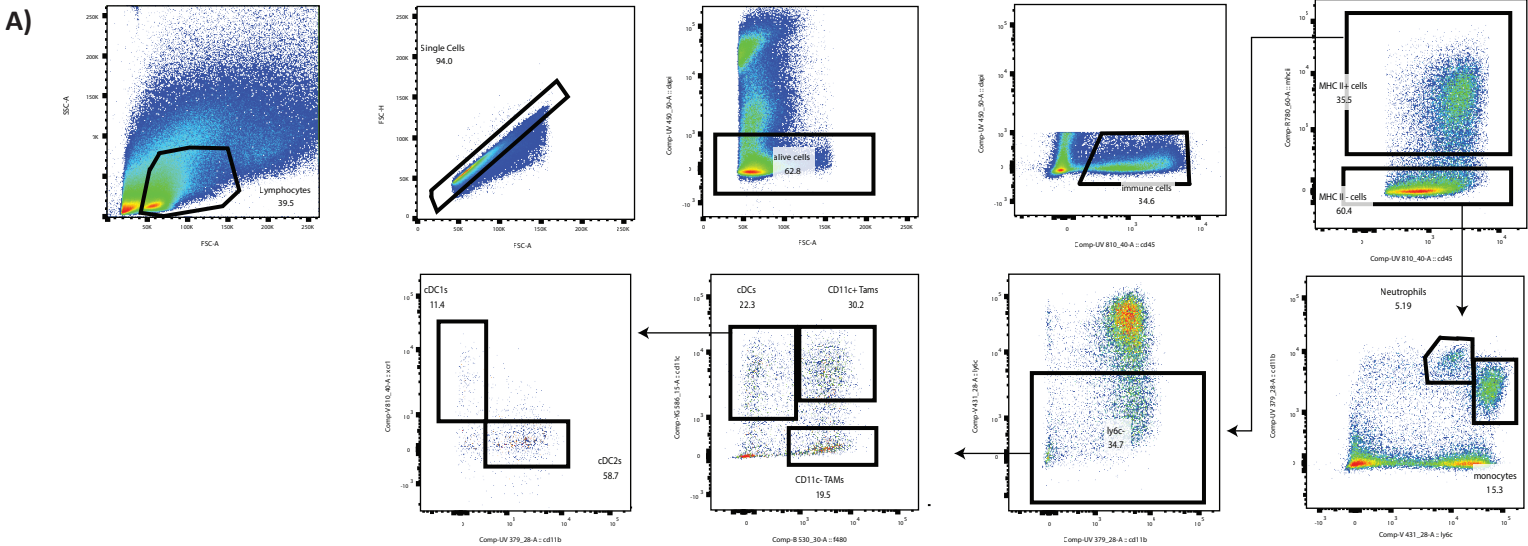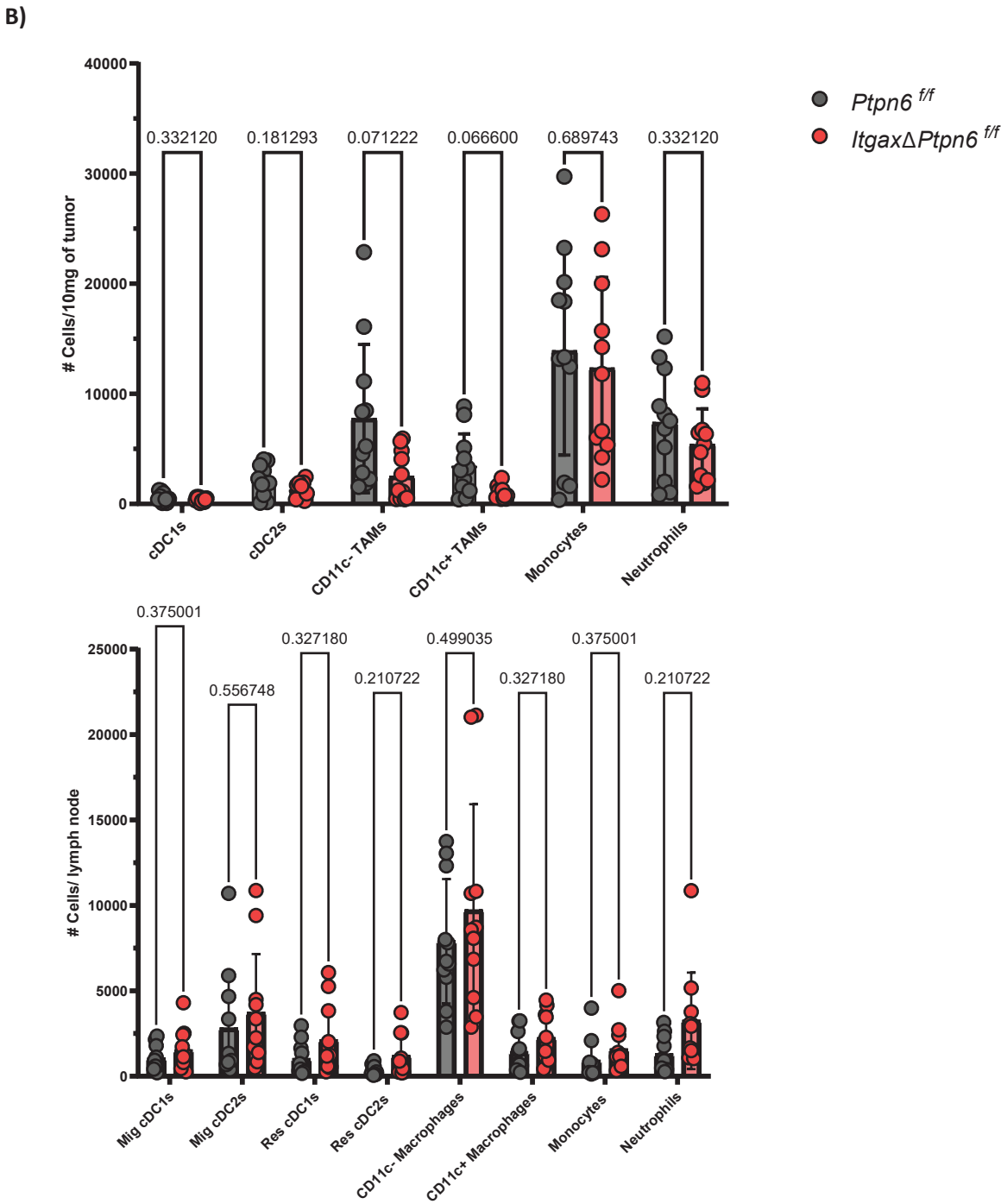

Figure S4

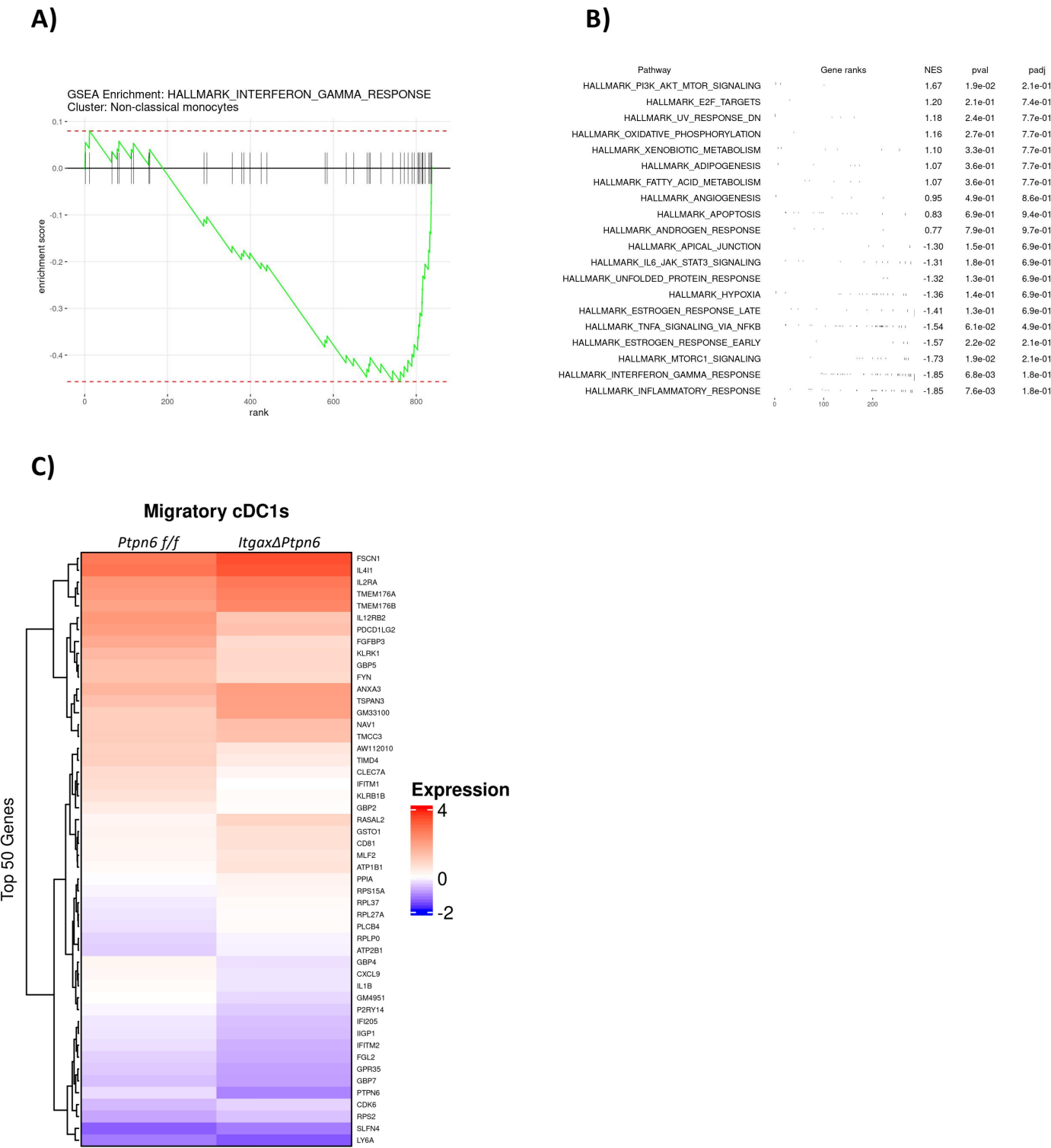

Figure S5

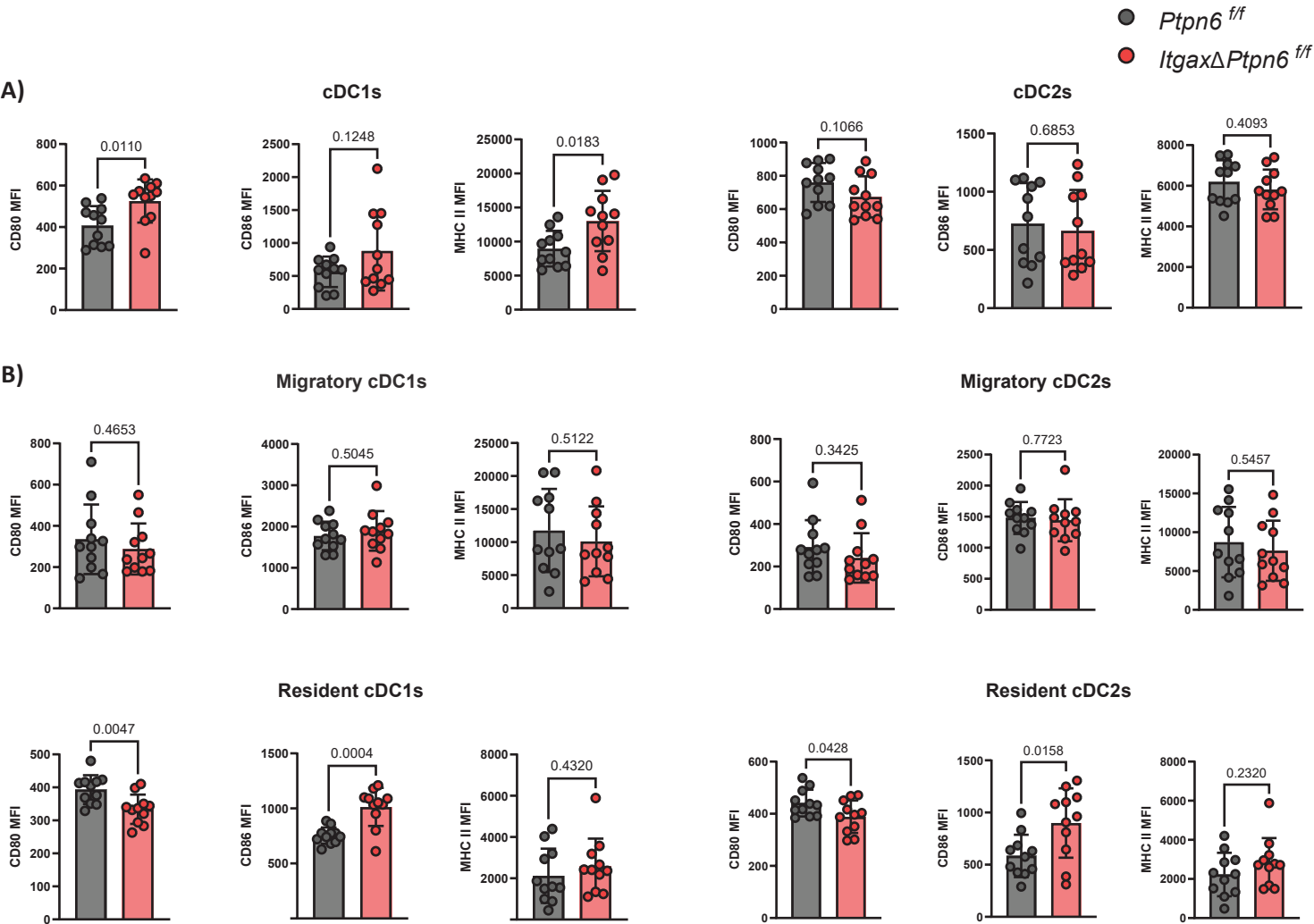

Figure S6

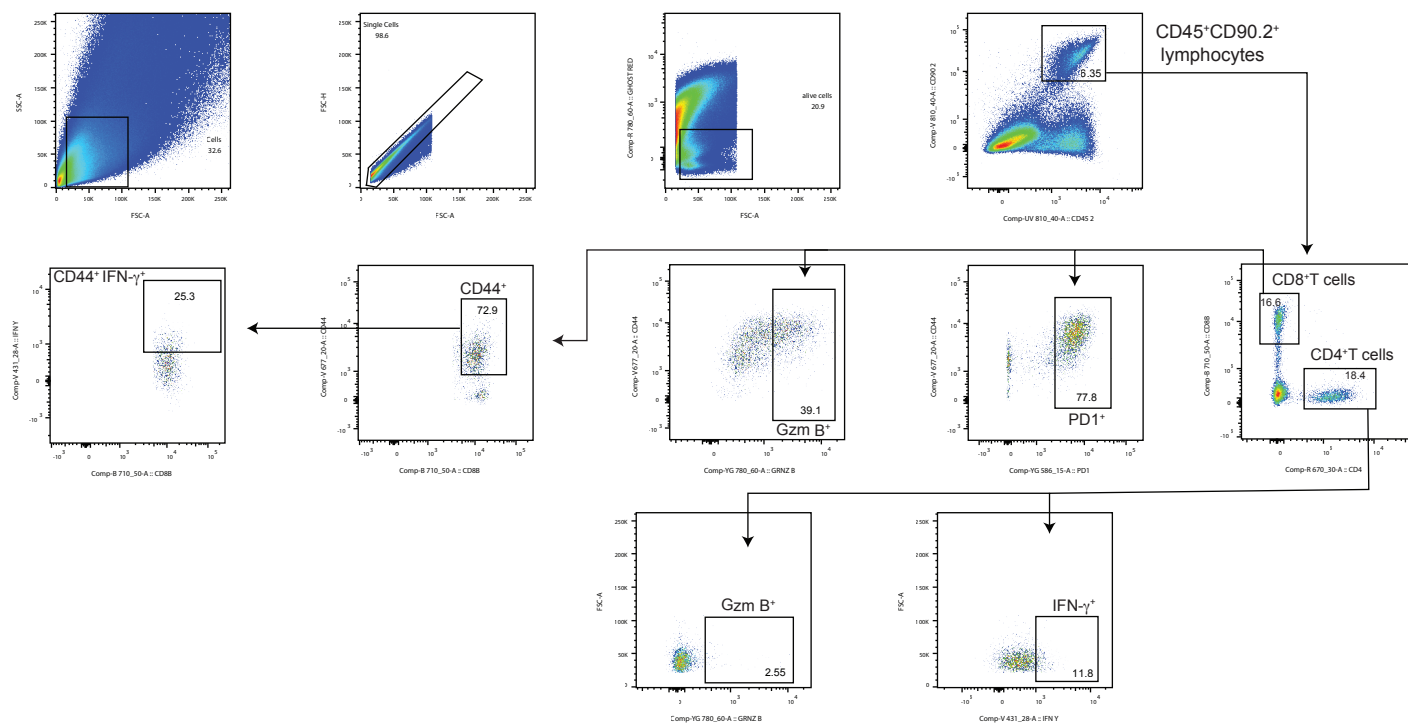

Figure S7

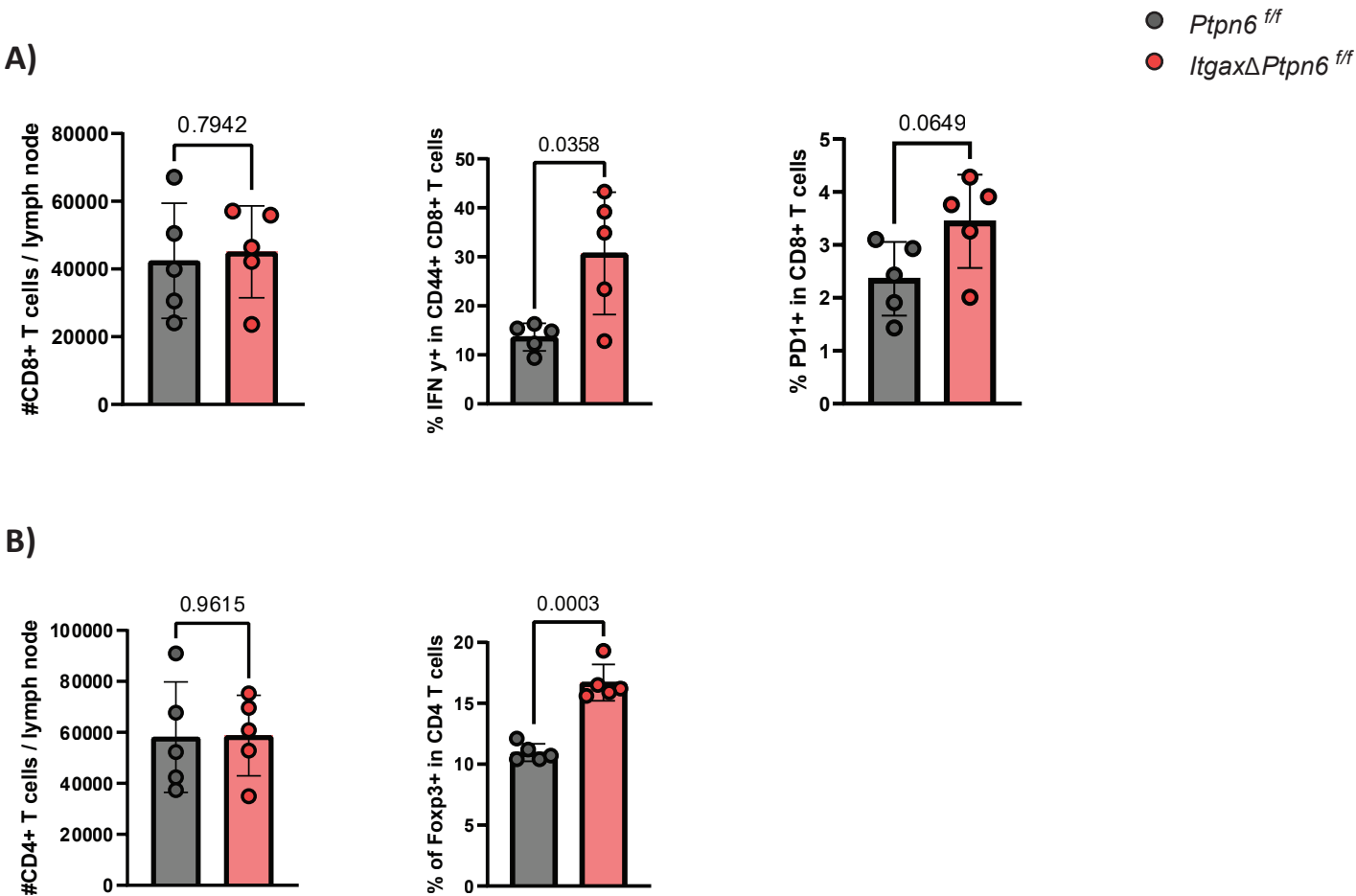

Supplement: Supplementary Figure S1 — Ptpn6 deletion does not affect Ptpn11 (SHP-2) mRNA expression in tumor-infiltrating myeloid cells. (A) Feature plots showing Ptpn6 expression across tumor-infiltrating CD11c+ myeloid cells identified by scRNA-seq from Ptpn6f/f and ItgaxΔPtpn6 mice bearing MC38 tumors. Expression levels are displayed on a UMAP projection, with colors indicating normalized transcript abundance. (B) Feature plots showing Ptpn11 (SHP-2) expression across the same cell populations. Each point represents a single cell. [file DataSheet1.pdf]
